# Pulmonary rehabilitation of a 72-year-old male with tracheostomy combined with unilateral tuberculous pleural effusion after cerebral infarction: A case report and literature review

**DOI:** 10.1097/MD.0000000000043360

**Published:** 2025-07-18

**Authors:** Haitang Wei, Ying Huang, Jun Yang, Tiecheng Guo, Lu Yang

**Affiliations:** a Department of Rehabilitation, Wuhan Hankou Hospital Affiliated to Wuhan University of Science and Technology, Wuhan, Hubei, China; b Department of Science and Education, Wuhan Hankou Hospital Affiliated to Wuhan University of Science and Technology, Wuhan, Hubei, China; c Department of Rehabilitation, Tongji Hospital Affiliated to Tongji Medical College, Huazhong University of Science and Technology, Wuhan, Hubei, China; d Hubei Province Key Laboratory of Occupational Hazard Identification and Control, Wuhan University of Science and Technology, Wuhan, Hubei, China; e Department of Rehabilitation, The Fifth Affiliated Hospital of Zhengzhou University, Zhengzhou, Henan, China.

**Keywords:** pleural effusion, pulmonary dysfunction, rehabilitation, stroke, tracheostomy

## Abstract

**Introduction:**

While poststroke rehabilitation primarily addresses motor, linguistic, cognitive, and swallowing impairments, pulmonary dysfunction (PD) is frequently neglected. PD following stroke, attributed to cortical-diaphragm pathway damage, can lead to increased mortality and prolonged hospitalization. Tracheostomy in such patients can exacerbate PD by increasing airway resistance and the risk of respiratory infections. This case study aims to report the successful integration of early pulmonary rehabilitation (PR) in a high-risk patient with poststroke tracheostomy complicated by unilateral tuberculous pleural effusion, underscoring its critical role in mitigating PD and improving outcomes.

**Patient concerns:**

A 72-year-old male with left-sided hemiplegia and dysphagia for over 3 months was admitted for rehabilitation following recurrent pulmonary infections post-cerebral infarction, which necessitated tracheostomy and indwelling tracheal cannula placement in the intensive care unit 3 months prior.

**Diagnoses:**

Cranial and thoracic computed tomography scans of the patient demonstrated infarctive lesions within the brainstem and the right semioval center, as well as evidence of infection in the lower lobe of the right lung. Additionally, atelectasis of the left lung and a significant amount of left-sided pleural effusion were observed. The patient’s T-cell spot test confirmed a positive result for tuberculosis infection. Due to the presence of dysphagia and bile reflux, a nasojejunal tube was inserted to facilitate enteral feeding. Furthermore, a tracheostomy was performed with the placement of an indwelling tracheostomy tube to manage respiratory difficulties. The patient was subsequently diagnosed with poststroke tracheostomy complicated by left-sided pleural effusion.

**Interventions:**

For this elderly patient who underwent tracheostomy following a cerebral infarction and subsequently developed pleural effusion, our team performed an integrated rehabilitation evaluation and treatment protocol, prioritizing PR strategies.

**Outcomes:**

The patient’s thoracic drainage tube and tracheostomy tube were successfully removed, with subsequent improvements in pulmonary function and overall motor function, leading to a reduction in the level of dependence on daily living activities.

**Lessons:**

For patients with pulmonary dysfunction following a stroke, PR should be considered an integral component of the rehabilitation plan. This approach is crucial for enhancing respiratory function, improving overall physical capacity, and thereby accelerating the recovery process.

## 1. Introduction

**Figure 1.**
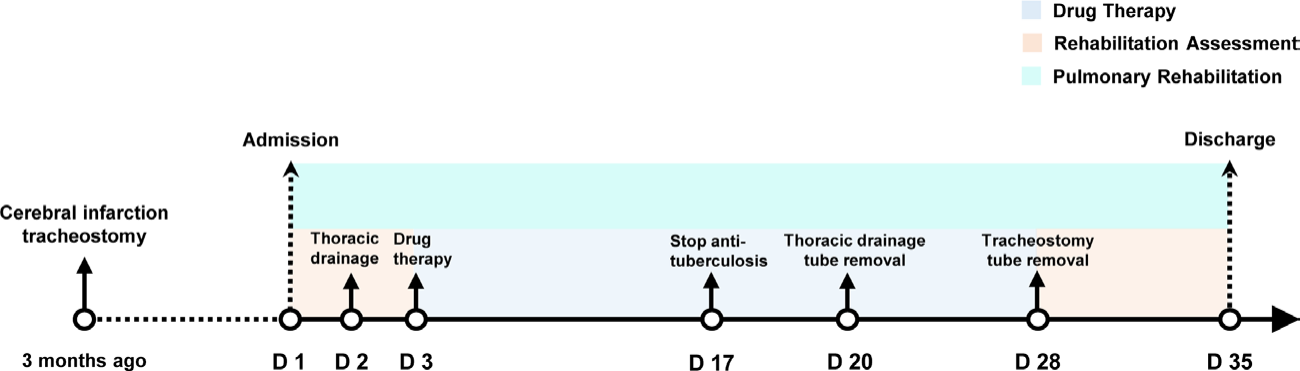
Schematic timeline of the patient’s illness onset and treatment process. The patient suffered a cerebral infarction 3 months ago and underwent a tracheostomy. Pulmonary rehabilitation lasted from admission to discharge (D1–D35). Rehabilitation assessments were performed post-admission (D1–D3) and predischarge (D28–D35). During D3–D28, disease-specific pharmacological treatment was administered, with anti-tuberculosis therapy discontinued on D17. Thoracic drainage was initiated on D2 and removed on D20, and the tracheal cannula was successfully extubated on D28.

The study of poststroke dysfunction is mainly focused on the recovery of patients’ motor function of hemiplegic limbs, language, cognition and swallowing function, but little attention has been paid to pulmonary dysfunction (PD).^[1]^ Ischemic or hemorrhagic stroke involving corticodiaphragmatic pathways disrupts supraspinal regulation of diaphragmatic contraction. Supratentorial (motor cortex/internal capsule) or brainstem lesions impair descending excitatory inputs to phrenic motor neurons (C3–C5), reducing diaphragm activation. This central paralysis induces hypoventilation, weak cough reflex, and aspiration risk due to compromised ventilation efficiency, emphasizing the critical role of corticodiaphragmatic integrity in post-stroke respiratory pathophysiology.^[2]^ And PD can also prolong hospital stay and increase the risk of death in patients.^[3]^ Tracheostomy is often used for artificial ventilation, sputum excretion and airway maintenance in critically ill patients, but the indwelling of tracheal cannula increases airflow resistance and respiratory work, reduces air humidification and heating, accelerates respiratory mucosal changes and leads to recurrent respiratory infection,^[4]^ which further aggravates PD in stroke patients.^[5]^ In addition, aspiration is often associated with swallowing dysfunction after tracheostomy, which is an important cause of aspiration pneumonia and delayed extubation in patients with tracheostomy after stroke.^[6]^ The main clinical manifestations of tuberculous pleural effusion (TBPE) include fever (86%), pleurisy-related chest pain (75%) and cough (70%), as well as other systemic symptoms^[7,8]^, and it often takes longer for elderly patients to develop symptoms.^[9]^ Presented herein is a rare case of a 72-year-old male patient who underwent tracheostomy after cerebral infarction, then complicated with unilateral TBPE. Early pulmonary rehabilitation (PR) was performed at the same time of drug treatment and pleural puncture drainage. Figure 1 illustrates the timeline of the patient’s onset of illness and treatment process.

## 2. Clinical data

### 2.1. Case profile

A 72-year-old male with a history of hypertension, partial gastrectomy, and chronic bile reflux developed left-sided hemiparesis, dysphagia with aspiration, and respiratory failure secondary to ischemic infarctions in the brainstem and right centrum semiovale. Persistent gastro-biliary reflux and vomiting necessitated nasojejunal tube placement. Concurrently, impaired cough reflex and prolonged immobilization precipitated recurrent pneumonia, requiring intensive care unit (ICU) admission and tracheostomy 3 months post-stroke.

### 2.2. Physical examination and auxiliary examination

On admission, the patient’s blood oxygen saturation was maintained at 96% under oxygen inhalation (oxygen flow 2 L/min) with an indwelling nasointestinal tube for infusion of nutrient solution. Auscultation of the right lung revealed diminished breath sounds and the presence of moist rale in the lower lobe, while the left lung exhibited significantly reduced breath sounds. The patient was conscious, alert, and able to follow medical instructions but experienced dysphagia and impaired speech. On physical examination, the left nasolabial fold was slightly shallow, and the tongue deviated to the left upon protrusion. Muscle tone was decreased on the left side, with Brunnstrom stages as follows: upper limb stage 2, hand stage 1, and lower limb stage 2. In contrast, the right limb demonstrated normal muscle strength and tone. The left Babinski sign was positive, while the Brudzinski sign was negative.

Laboratory tests revealed a red blood cell count of 3.8 × 10^12^/L, a hemoglobin level of 97 g/L, and an albumin level of 26 g/L. Inflammatory markers were elevated, with a D-dimer level of 3.29 μg/mL, interleukin-6 at 51.6 pg/mL, and a hypersensitive C-reactive protein level of 74.2 mg/L. Pleural effusion analysis showed a red hue, with a cell count of 4520 × 10^6^/L, a lymphocyte percentage of 12%, lactate dehydrogenase at 196 U/L, and total protein at 53.3 g/L. Culture of the pleural effusion yielded no bacterial growth, and the DNA result for the pleural effusion TB complex group was negative. Bronchial lavage fluid and fiberoptic bronchoscope brush smears for TB were also negative, as was the PPD test (5 × 5 mm). However, the tuberculosis infection T-cell spot test was positive.

**Figure 2.**
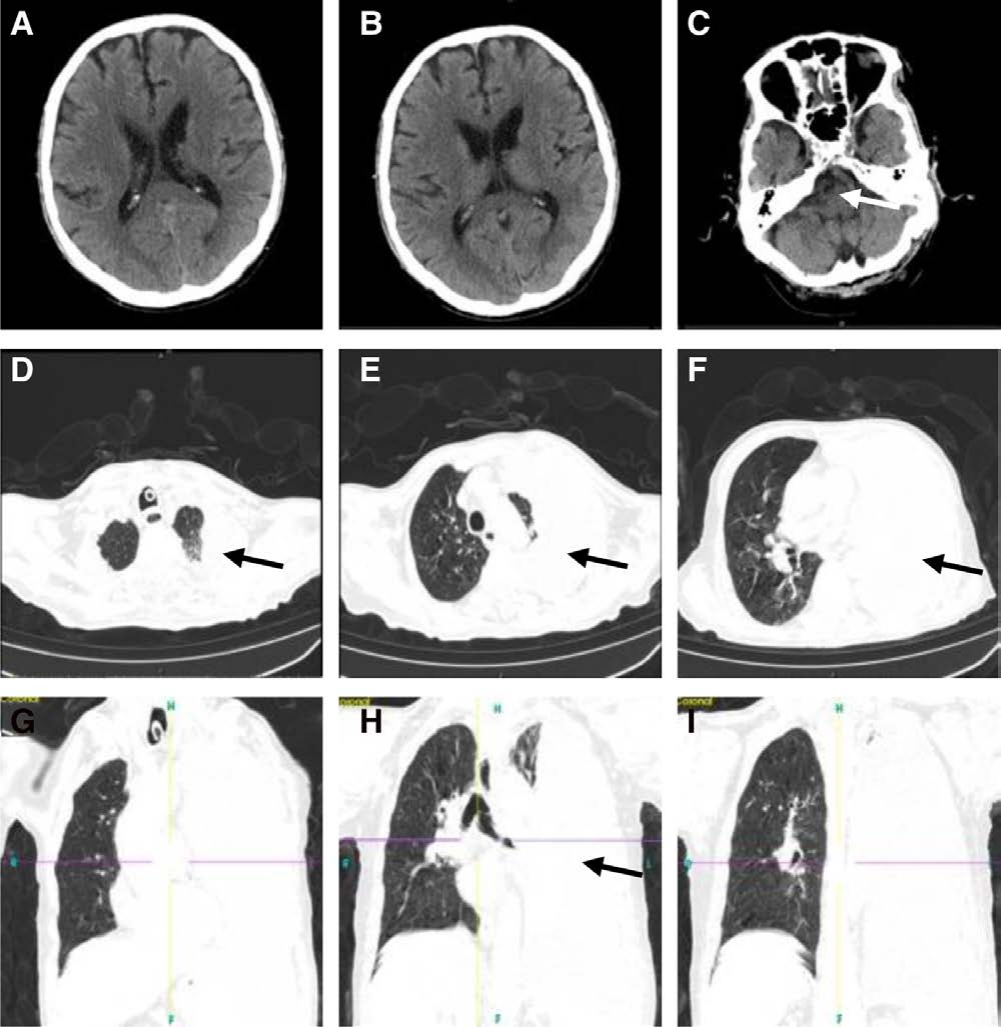
Cranial and thoracic CT scans after admission. Cranial CT (A–C) revealed cerebral infarction in the basal ganglia and the brainstem, along with a right hemioval center softening lesion, causing hemiplegia, dysphagia, and pulmonary dysfunction; thoracic CT (D–I) showed left pleural effusion, causing dyspnea. CT = computed tomography, PD = pulmonary dysfunction.

Cranial and thoracic computed tomography scans post-admission (Fig. 2) demonstrated bilateral lacunar infarctions in the basal ganglia and brainstem, along with a right hemioval center softening lesion and cerebral atrophy. In the lungs, there was evidence of infection in the lower lobe of the right lung, bronchial occlusion of the left lung, atelectasis of the left lung, and a significant pleural effusion in the left thoracic cavity. Additionally, there were a few areas of fibrous induration and proliferation in the dorsal segments of both upper and lower lobes.

### 2.3. Diagnosis

Based on the patient’s medical history, past medical history, complaints, physical examination, laboratory tests, and imaging studies, the patient was diagnosed as follows: 1. cerebral infarction convalescence stage with existing dysfunction: left limb motor dysfunction; PD; swallowing dysfunction; balance and walking dysfunction; activities of daily living severe dependence according to the International Classification of Functioning, Disability and Health; 2. extubation difficulties after tracheostomy; 3. aspiration pneumonia; 4. left TBPE with left atelectasis; 5. hypertension of grade 3 with very high risk.

## 3. Treatment

### 3.1. Drug therapy

Post-hospitalization, the patient was under continuous cardiac monitoring for blood pressure and oxygen saturation. Enteral feeding was conducted through a nasojejunal tube with infusion pump support. A multidisciplinary team from the ICU and respiratory unit was consulted for further management. The treatment regimen encompassed anti-inflammatory therapies, antituberculosis treatment, mucolytic agents, cerebral nutrition support, antihypertensive medications, gastric protection, prokinetic drugs, albumin replacement, and antituberculosis therapy.

### 3.2. Thoracic puncture and drainage

**Figure 3.**
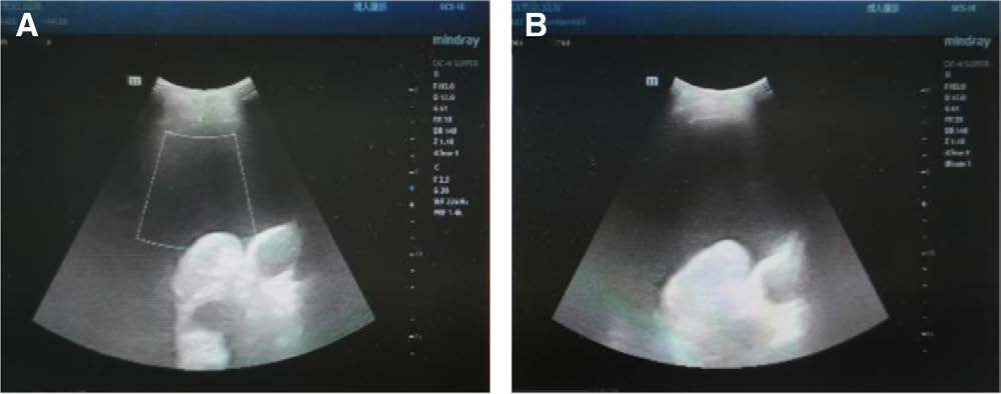
Ultrasound examination of pleural effusion. (A and B) Ultrasound examination indicated a large amount of pleural effusion on the left side.

Upon admission, the patient underwent ultrasound-guided thoracentesis for the drainage of pleural effusion.^[10]^ A large pleural effusion with an anteroposterior diameter of approximately 108 mm was observed in the left hemithorax, with significant floating lung lobes noted within the fluid collection. The depth of the effusion from the body surface at the puncture site was approximately 16 mm. Ultrasound examination is depicted in Fig. 3.

### 3.3. Rehabilitation evaluation and treatment

Upon admission, the patient underwent a comprehensive rehabilitation assessment, which included the modified Medical Research Council (mMRC) Dyspnea Scale, Borg Dyspnea Score, and pulmonary function testing^[11,12]^ (Fig. S1, Supplemental Digital Content, https://links.lww.com/MD/P402) using the Racer (Xiamen) Pulmonary Function Tester (Model: X1). The testing indicators comprised forced vital capacity (FVC), forced expiratory volume in one second (FEV_1_), peak expiratory flow (PEF), the ratio of FEV_1_ to FVC, maximal inspiratory pressure (MIP), and maximal expiratory pressure (MEP). Additional assessments included a cough symptom score sheet, sputum character classification, cough force grading, sputum volume grading, Modified Barthel Index, Fugl-Meyer Assessment, DE Morton Mobility Index,^[13]^ Self-rating Anxiety Scale (SAS), and Self-rating Depression Scale (SDS). Based on the evaluation of functional impairments, an individualized comprehensive rehabilitation treatment plan focusing on PR was formulated.

*Motor function training*: To enhance cardiorespiratory fitness and augment the strength and endurance of muscle groups in the limbs and trunk, a regimen comprising comprehensive training for hemiplegic limbs, bridge exercises, rolling exercises, sitting exercises, and electric tilt table training was employed. This intervention was also aimed at alleviating symptoms of respiratory distress and elevating the patient’s exercise endurance.

**Figure 4.**
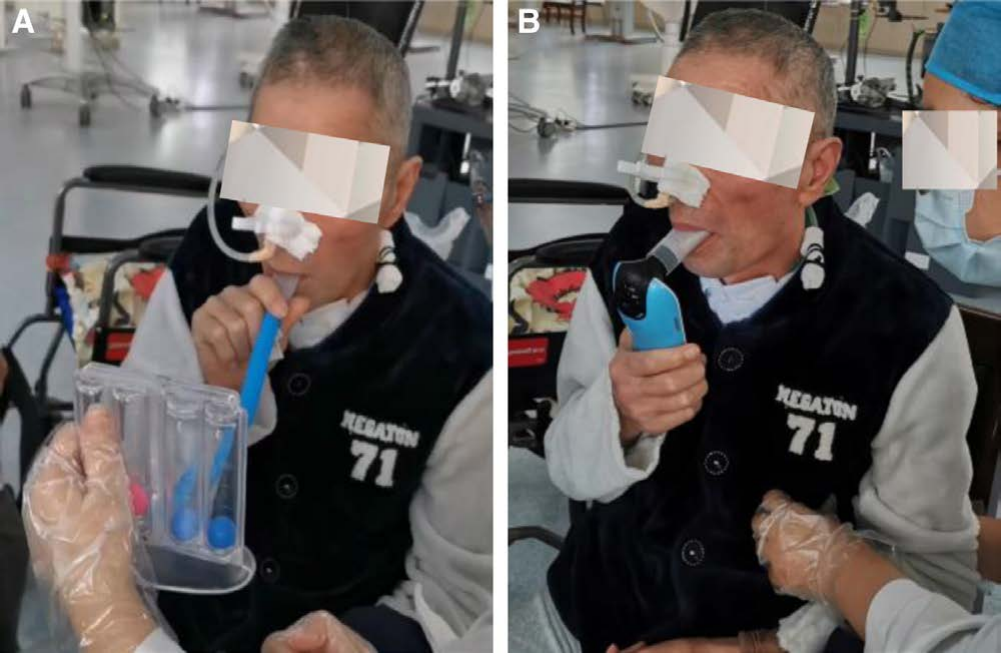
Pulmonary rehabilitation training of the patient. (A) Resistance training for inspiratory and expiratory muscles using a dual-valve breathing trainer; (B) exhalation positive airway pressure (EPAP) combined with high-frequency oscillation (HFO) training.

*PR training*: This program was aimed at improving pulmonary function, managing pulmonary infections, and hastening the evacuation of pleural effusions. *Postural management*. For this patient with left-sided pleural effusions, semi-seated or left lateral positions were adopted to facilitate ventilation in the healthy lung. And a gradual progression in postural training was implemented, transitioning from sitting with support to independent sitting, followed by assisted standing, and ultimately to unsupported standing. *Respiratory training* (see Video, Supplemental Video 1, which demonstrates part of the training). This training was beneficial for strengthening respiratory muscle strength and endurance, improving cough efficacy, and ameliorating pulmonary ventilation. During the training, the tracheostomy tube opening was temporarily sealed with medical tape, and vigilant monitoring of the patient’s complexion, mental status, heart rate, oxygen saturation, and respiratory distress was conducted. Each session lasted for 20 minutes, twice daily. Abdominal breathing and pursed-lip breathing were practiced with an inspiratory to expiratory ratio ranging from 1:2 to 1:3 in order to reestablish respiratory pattern. Resistance training for inspiratory and expiratory muscles using a dual-valve breathing trainer (Fig. 4A), with incremental increases in respiratory resistance based on the patient’s tolerance and adaptability during the training sessions. *Airway clearance therapy*. This treatment aided in the expectoration of sputum, reduced airway obstruction and mitigated pulmonary infection. Conducted for 30 minutes per session, twice daily. The protocol consisted of the following techniques: Cough training (Fig. S2A, Supplemental Digital Content, https://links.lww.com/MD/P402). The patient was guided to take a deep inspiration, hold breath for 1 to 2 seconds, and then coughed vigorously. The therapist applied manual abdominal compression during exhalation to enhance intra-abdominal pressure. *Manual chest vibration*. The therapist’s hands are positioned with fingers together and the palm cupped, delivering alternate percussive claps to the chest wall over the targeted drainage area. *Active cycle of breathing techniques*. The patient performed his natural pattern of tidal breathing, followed by a voluntary breath hold at the end of inhalation for 3 seconds, and then forceful exhalation 1 to 2 times. Exhalation positive airway pressure combined with high-frequency oscillation training (Fig. 4B). High-frequency oscillation aided in the mobilization of secretions, while exhalation positive airway pressure supported lung expansion and prevented airway collapse. Utilizing a portable pulmonary function device, the airway pressure was incrementally adjusted from 3 to 12 cm H_2_O based on the patient’s tolerance. (4) *Phrenic nerve pacing*. A Jilin Dia Health B variable frequency portable phrenic nerve pacemaker (Fig. S2B, Supplemental Digital Content, https://links.lww.com/MD/P402) was used. The patient was positioned in a semi-seated posture, with the 2 main electrode pads placed below one-third the outer edge of the sternocleidomastoid muscle on both sides, and the 2 auxiliary electrode pads placed at the intersection of the midclavicular line with the second rib on both sides. The pacing frequency was set at 9 to 15 cycles per minute, with a stimulation intensity of 12 to 30 units, for a duration of 20 minutes per session, twice daily. This therapy was beneficial for enhancing the strength and endurance of the diaphragm, increasing diaphragmatic mobility, and thereby improving pulmonary ventilation and gas exchange (File S1, Supplemental Digital Content, https://links.lww.com/MD/P404 provides more details about PR).

*Swallowing function rehabilitation*: a comprehensive program designed to enhance deglutition capabilities includes the following strategies: *maxillofacial and oromotor exercises*. Including training of the jaw, lips, and tongue muscles, with therapy sessions conducted twice daily. *Sensory stimulation training*. Utilizing a tongue depressor or cotton swabs dipped in ice water or fruit juice to stimulate the perioral area, tongue, and posterior pharyngeal wall, administered twice daily. *Neuromuscular electrical stimulation*. A low-frequency electrical stimulation of the swallowing muscles was applied using the Fitzmann VOCA STIM-Master device (German), with each session lasting 20 to 30 minutes, conducted twice daily. (4) Therapeutic feeding exercises. Based on the outcomes of dye tests and videofluoroscopic swallowing assessment (Fig.S3, Supplemental Digital Content, https://links.lww.com/MD/P402), which indicated this patient’s aspiration with more than 4ml of food type 1, and residued in the vallecula and piriform sinus with complete clearance after 3 swallows of food type 1 to 3, the appropriate food consistency was selected, and swallowing techniques were integrated to reduce the risk of aspiration. These exercises were carried out for 20 minutes per session, once daily.

*Psychological intervention*: Upon admission, the patient underwent assessment using the SAS and SDS, which indicated moderate anxiety and depression. The patient expressed a loss of confidence in treatment, exhibited suicidal ideation, and demonstrated poor engagement and cooperation with the therapeutic process. Through communications with the patient and his family, increased family support and accompaniment by medical staff were provided, along with education on disease-related knowledge and health advocacy. This approach aimed to alleviate the patient’s fear of the illness and bolster confidence in combating the disease, thereby improving the patient’s anxiety and depressive symptoms.

### 3.4. Comprehensive rehabilitation assessment of therapeutic efficacy

**Figure 5.**
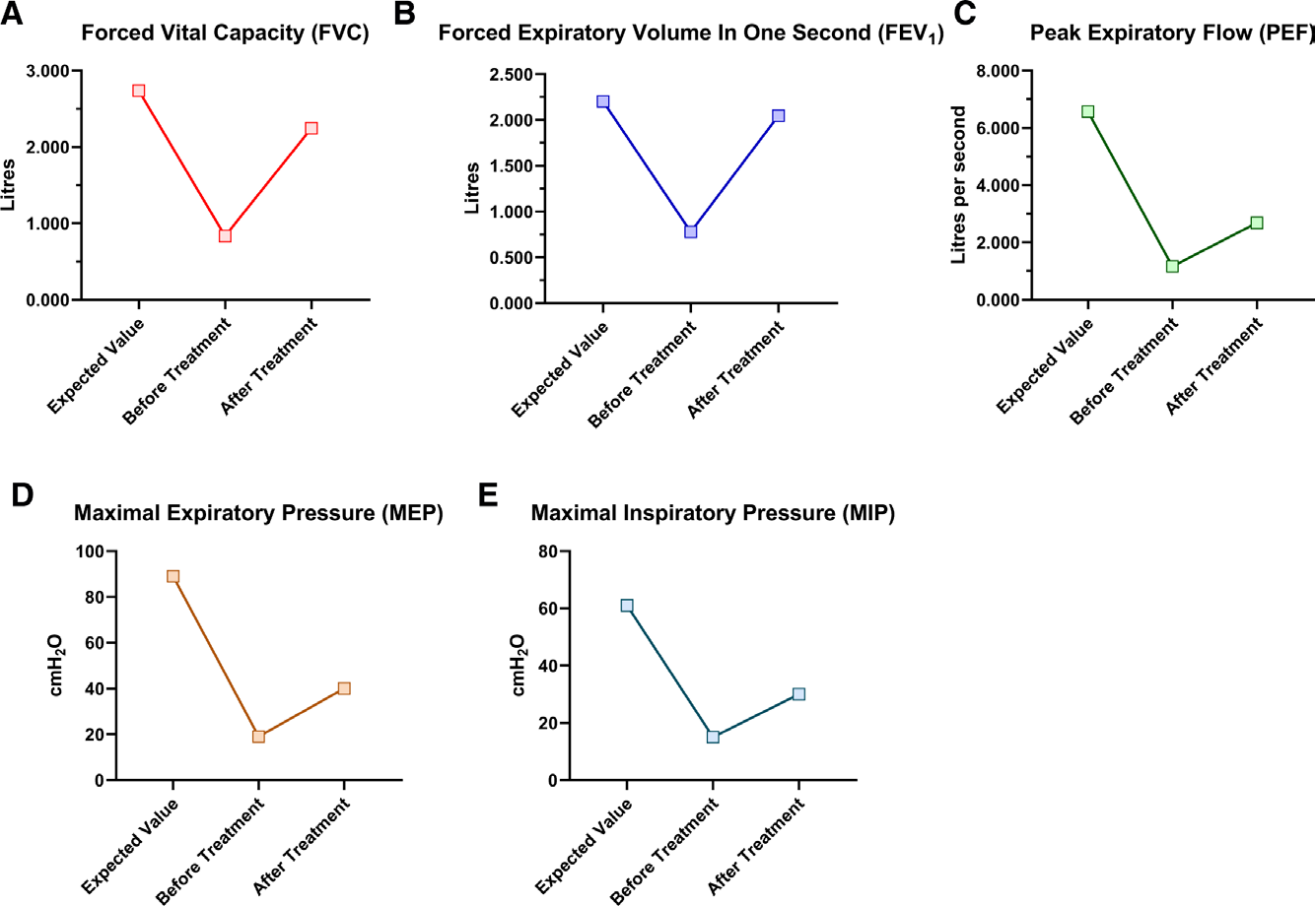
The values of FVC, FEV_1_, PEF, MEP, and MIP before and after treatment. (A) Patient’s expected FVC and FVC values before and after treatment; (B) patient’s expected FEV_1_ and FEV_1_ values before and after treatment; (C) patient’s expected PEF and PEF values before and after treatment; (D) patient’s expected MEP and MEP values before and after treatment; (E) patient’s expected MIP and MIP values before and after treatment. FEV1 = forced expiratory volume in one second, FVC = forced vital capacity, MIP = maximal inspiratory pressure, MEP = maximal expiratory pressure, PEF = peak expiratory flow.

Following 35 days of individualized rehabilitation, the patient exhibited significant functional improvements across domains:

*Pulmonary function and respiratory outcomes*:

[1] Dyspnea severity decreased: mMRC score improved from 4 to 3; Borgs score reduced from 5 to 3.
[2] Pulmonary function gains: FVC increased from 0.833 to 2.245 L; FEV1 rose from 0.778 to 2.046 L; peak expiratory flow elevated from 1.161 to 2.686 L/s; MIP improved from 15 to 30 cm H₂O; MEP from 19 to 40 cm H₂O (Table 1 and Fig. 5).
[3] Cough efficacy enhanced: frequency declined from occasional daytime coughing to no nocturnal coughs; the sputum consistency score advanced from P1 (one-third purulent, two-thirds mucoid) to M1 (mucopurulent sputum without purulence); cough force upgraded from grade 2(weak cough sound) to grade 3(clear cough sound); sputum volume reduced (from moderate to small) (Table 2); the respiratory rate reduced from 24 to 26 bpm to 18 to 20 bpm (Table 4).

**Table 1:** Comparison of respiratory function assessment before and after patient treatment.

Assessment Time	mMRC score	Borgs score	FVC	FEV_1_	FEV_1_/FVC ratio	PEF	MEP	MIP
Before treatment	4	5	0.833 L	0.778 L	93.3%	1.161 L/S	19 cm H_2_O	15 cm H_2_O
After treatment	3	3	2.245 L	2.046 L	91.1%	2.686 L/S	40 cm H_2_O	30 cm H_2_O

The expected values are as follows: FVC = 2.739L, FEV_1_ = 2.204 L, PEF = 6.572 L/S, MEP = 89 cm H_2_O, MIP = 61 cm H_2_O. FEV1 = forced expiratory volume in one second, FVC = forced vital capacity, mMRC = modified Medical Research Council, MIP = maximal inspiratory pressure, MEP = maximal expiratory pressure, PEF = peak expiratory flow.

**Table 2:** Comparison of cough and sputum assessment before and after patient treatment.

Assessment time	Cough symptom score	Sputum consistency score	Cough force grade	Sputum volume grade
Before treatment	Day 1/Night 1 (occasional brief cough)	P1 (1/3 purulent, 2/3 mucoid)	2 (weak cough sound)	Moderate (10–150 mL/day)
After treatment	Day 1/Night 0 (no cough)	M1 (mucoid, no purulence)	3 (clear cough sound)	Small (<10 mL/day)

**Figure 6.**
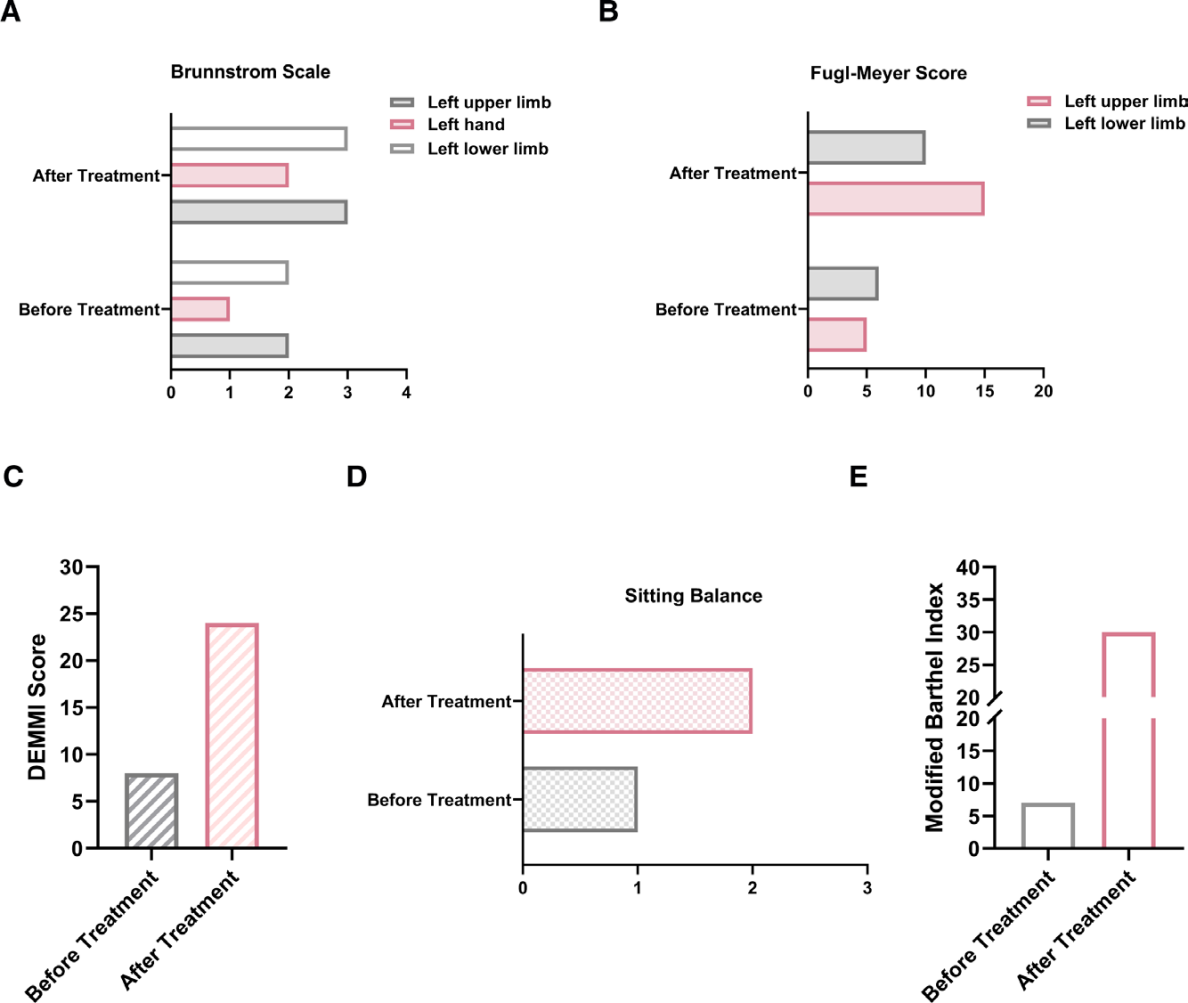
The results of motor function assessment before and after treatment. (A) The patient’s Brunnstrom Scale (left upper limb, hand, and lower limb) scores before and after treatment; (B) the patient’s Fugl-Meyer Scale scores before and after treatment; (C) the patient’s DEMMI scores before and after treatment; (D) the evaluation results of Sitting Balance before and after treatment; (E) the evaluation results of Modified Barthel Index before and after treatment. DEMMI = DE Morton mobility Index.

*Motor function and mobility*:

[1] Achieved independent sitting (sitting balance: improved from grade 1 to grade 2).
[2] Limb function: Brunnstrom scale: left upper limb: improved from 2 to 3; hand: improved from 1 to 2; lower limb: improved from 2 to 3. Fugl-Meyer score: upper limb: improved from 5 to 15; lower limb: improved from 6 to 10.
[3] Mobility: DE Morton mobility Index score: improved from 8 to 24; modified Barthel Index: improved from 7 to 30 (indicating reduced activities of daily living dependency) (Table 3; Fig. 6).

**Table 3:** Comparison of motor function assessment before and after patient treatment.

Assessment time	Brunnstrom scale	Fugl-Meyer score	DEMMI score	Sitting balance	Modified Barthel Index
Before treatment	Left upper limb 2, hand 1, lower limb 2	Upper limb 5, lower limb 6	8	1	7 (completely dependent)
After treatment	Left upper limb 3, hand 2, lower limb 3	Upper limb 15, lower limb 10	24	2	30 (severely dependent)

Fugl-Meyer score: maximum of 66 points for the upper limbs, maximum of 34 points for the lower limbs. DEMMI score: maximum of 100 points. Sitting balance grade: maximum of 3 grade.

DEMMI = DE Morton mobility index.

**Figure 7.**
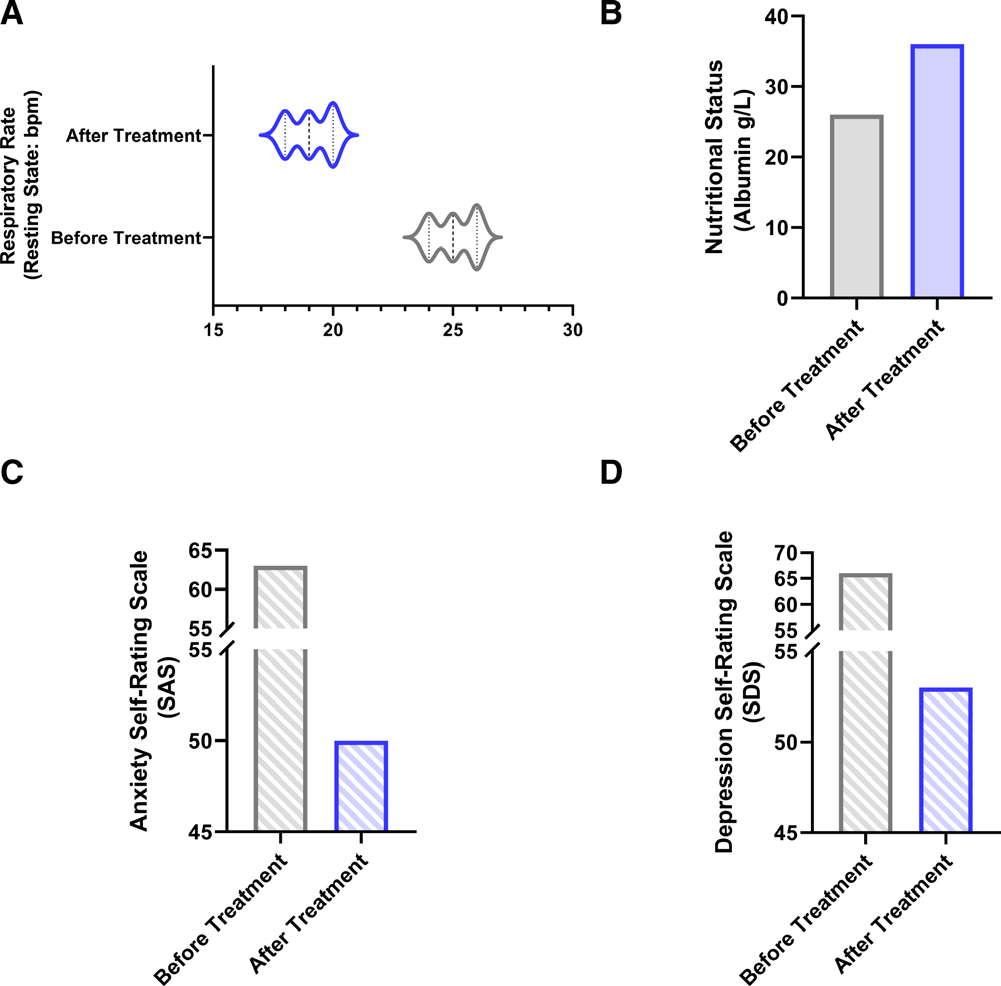
The values of overall condition assessment before and after treatment. (A) The patient’s respiratory rate (resting state) values before and after treatment; (B) the patient’s albumin levels (g/L) before and after treatment; (C) the patient’s Anxiety Self-Rating Scale (SAS) scores before and after treatment; (D) the patient’s Depression Self-Rating Scale (SDS) scores before and after treatment.

*Nutritional parameters*: Albumin increased from 26.0 to 36.0 g/L (Table 4; Fig. 7).

*Psychological status*: SAS score: from 63 to 50 (improved from moderate to mild anxiety); SDS score: 66 to 53 (improved from moderate to mild depression) (Table 4; Fig. 7).

**Table 4:** Comparison of overall condition assessment before and after patient treatment.

Assessment time	Oxygen saturation (resting state)	Respiratory rate (resting state)	Nutritional status (albumin)	Anxiety self-rating scale (SAS)	Depression self-rating scale (SDS)
Before treatment	96% (on oxygen)	24–26 bpm	26.0 g/L	63 (moderate anxiety)	66 (moderate depression)
After treatment	95–96% (without oxygen)	18–20 bpm	36.0 g/L	50 (mild anxiety)	53 (mild depression)

**Figure 8.**
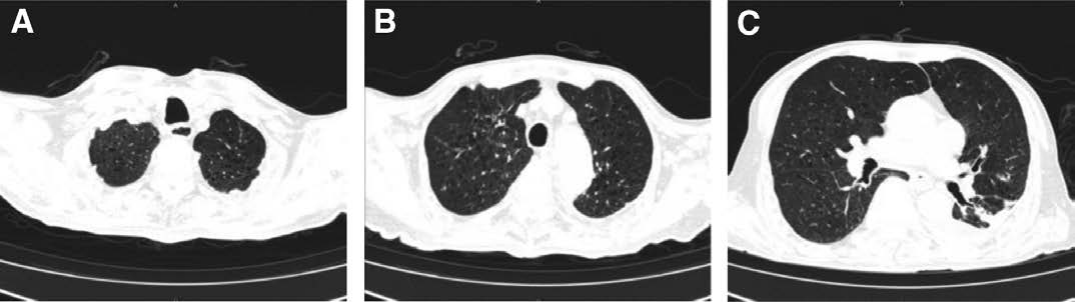
Thoracic CT scan following the removal of the pleural effusion catheter and tracheostomy tube from the patient. (A–C) The images indicated significant improvement in the patient’s pleural effusion and pulmonary infection. CT = computed tomography.

*Clinical milestones*: thoracic drainage tube removed on day 20 (post-effusion resolution); tracheostomy decannulated on day 28 (Fig. 8); oxygen saturation maintained 95% to 96% without supplemental O_2_ (Table 4).

In addition to thoracic drainage and pharmacotherapy, the patient began an integrated rehabilitation program focused on PR soon after admission. After the pleural effusion had subsided, the thoracic drainage tube was successfully removed. Subsequently, on day 28 of hospitalization, the patient fulfilled the indications for tracheostomy tube removal and the tube was smoothly removed.

## 4. Discussion

Studies have demonstrated that PR can improve exercise capacity, health-related quality of life, and ameliorate symptoms of dyspnea, fatigue, anxiety, and depression in patients with chronic respiratory diseases.^[14]^ However, a mere 5% of eligible patients actually undergo PR,^[15]^ reflecting systemic barriers such as limited healthcare access, insufficient clinician awareness, and fragmented referral pathways. PR after exacerbation of chronic obstructive pulmonary disease is effective in reducing chronic obstructive pulmonary disease hospitalizations and mortality while improving health-related quality of life. The clinical adoption and dissemination of pulmonary rehabilitation remain a significant challenge despite robust evidence of its cost-effectiveness: a 2022 economic evaluation demonstrated that PR resulted in net cost savings per patient of $5721 (95% prediction interval, $3307–$8388) and improved quality-adjusted life expectancy (gain of 0.53 years [95% prediction interval, 0.43–0.63]).^[16]^ This case illustrates the clinically significant potential of pulmonary rehabilitation in a high-risk patient with poststroke tracheostomy and pleural effusion, whose functional status improved markedly following a tailored regimen.

Stroke patients often experience respiratory dysfunction, with a decrease in central respiratory drive and respiratory reserve capacity. Primary central nervous system damage or secondary complications from stroke can lead to weakened respiratory muscles, particularly central diaphragmatic paralysis due to impairment of the cortico-diaphragmatic pathway.^[2]^ This results in reduced diaphragmatic excursion, with FVC and FEV1 decreasing to 50% of predicted values,^[17]^ and MIP and MEP being reduced by more than 50%.^[18]^ Furthermore, the weakened diaphragm and intercostal muscles on the paralyzed side lead to asymmetric thoracic wall movement and abnormal breathing patterns, which decrease tidal volume and exercise endurance in these patients.^[19]^ Liu X et al^[20]^ reported that the incidence of diaphragmatic dysfunction during the recovery period in stroke patients with hemiplegia is 46.67%, with a significant decrease in diaphragmatic activity and thickness fraction during deep breathing. The diaphragm, being the most crucial inspiratory muscle, is intimately linked to pulmonary function. Strengthening the diaphragm can increase intrathoracic negative pressure, thereby enhancing lung compliance, reducing airway resistance, and improving pulmonary ventilation and gas exchange efficiency.^[21]^ Electrical stimulation of the phrenic nerve induces rhythmic contractions of the diaphragm, and B Yan et al^[22]^ discovered that the use of an external diaphragmatic pacemaker combined with oxygen therapy can improve pulmonary function and diaphragmatic excursion in patients post-intracerebral hemorrhage, also reducing ICU stay and hastening the weaning from mechanical ventilation. Additionally, several studies have shown that respiratory muscle training (RMT) can increase respiratory muscle strength, enhance cough capacity, reduce the incidence of stroke-associated pneumonia, and improve swallowing function in stroke patients.^[23]^ Also, a randomized controlled trial conducted by Choi et al demonstrated that implementing a 4-week intensive RMT protocol in patients with acute stroke resulted in significantly greater improvements in both pulmonary function and respiratory muscle strength than standard rehabilitation. These findings suggest that early integration of RMT may serve as a promising adjunctive intervention to mitigate respiratory complications.^[24]^

Respiratory dysfunction in patients with tracheostomy after stroke is not only related to the stroke itself but is also significantly influenced by the tracheostomy procedure. Tracheostomy can disrupt the normal swallowing process, leading to reduced laryngeal elevation, decreased sensitivity and cough reflex in the larynx, absence of subglottic pressure, and poor coordination between swallowing and breathing. These disruptions can exacerbate dysphagia in stroke patients, leading to aspiration and the development of aspiration pneumonia. Research has demonstrated a strong correlation between the decline in pulmonary function in stroke patients and the frequency of aspiration episodes.^[25]^ Enhancement of pulmonary function can mitigate aspiration risks, which in turn can lower the incidence of aspiration pneumonia, ameliorate pulmonary infections, and facilitate earlier decannulation and increased decannulation success rates. In this case, the patient presented with stroke complicated by tracheostomy and left-sided pleural effusion, exhibiting both dysphagia and respiratory dysfunction, with weakened respiratory muscles including the diaphragm. The patient also experienced reflux and aspiration, culminating in aspiration pneumonia. A comprehensive pulmonary rehabilitation program that included respiratory muscle training, airway clearance techniques, and external diaphragmatic pacing, along with targeted swallowing function exercises, led to improvements in pulmonary function and reduced aspiration. This approach not only alleviated pulmonary infections but also supported earlier removal of the tracheostomy tube and hastened the patient’s recovery process.

In the management of pleural effusion, thoracic drainage aids in rapidly alleviating symptoms such as dyspnea and pleuritic pain in patients.^[9]^ However, the presence of a drainage tube not only causes additional pain but also leads to an increased risk of thoracic drainage-related complications, which range from 4.8% to 22.3%,^[26]^ with the incidence rising as the duration of thoracic drainage extends.^[27]^ The most common complications include tube blockage, followed by pleural space infection (empyema), catheter malposition, and injury to intrathoracic tissues. Therefore, minimizing the duration of indwelling pleural catheters^[28]^ and promoting expeditious drainage of pleural effusion are crucial therapeutic objectives.^[12,29]^ Although early scholars have suggested incorporating physical therapy into respiratory rehabilitation programs for the treatment of pleural effusion,^[30,31]^ a review of the literature reveals a scarcity of recent studies in this aera.Pleural effusion can result in reduced lung capacity and restricted expansion of the chest wall,^[32]^ and the rehabilitation focuses on addressing dyspnea, localized pain, inefficient breathing patterns, activity intolerance, and anxiety. Evidence indicates that respiratory rehabilitation training can improve respiratory function and mitigate the risks associated with intensive care and bed rest, even in patients with severe functional impairments.^[10]^ In a clinical randomized controlled study on pleural effusion by Valenza-Demet G et al,^[10]^ the control group received standard treatment (medical treatment and drainage), while the intervention group received physical therapy in addition to the control group’s treatment (deep breathing exercises, physical training). Posttreatment, the intervention group showed significant improvements in FVC, FEV1 and FEF_25–75_ (forced expiratory flow between 25% and 75% of vital capacity) compared to the control group. Moreover, the intervention group had better radiological scores for pleural effusion on discharge, and their hospital stay was significantly shorter (26.7 ± 8.8 days) than that of the control group (38.6 ± 10.7 days) (*P* = .014). This study suggests that integrating a physical therapy regimen with standard treatment can enhance spirometric parameters and radiological outcomes in patients with pleural effusion, expedite the drainage process, decrease the incidence of catheter-related complications, and reduce hospital stay durations. In this case, the patient, managed with medical treatment and thoracic drainage, benefited from early and aggressive pulmonary rehabilitation, which not only shortened the drainage time for pleural effusion and lowered the risk of catheter-related complications but also enhanced pulmonary function and overall exercise performance.

On the other hand, studies have reported that 50% of patients with pleural tuberculosis develop pleural thickening after 1 year of antituberculosis medication.^[33]^ Additionally, Chung CL et al^[34]^ have observed that a swift decline in the volume of pleural effusion may correlate with a reduced occurrence of pleural fibrosis. It is significant to note that rehabilitation therapy, by accelerating the drainage of pleural effusion and improving PD, may potentially contribute to a decrease in the incidence of pleural fibrosis. Thus, the active integration of rehabilitation treatment could be instrumental in mitigating the risk of pleural fibrosis.

## 5. Limitations

While this case demonstrated the efficacy of integrated pulmonary rehabilitation in a complex poststroke scenario, several limitations warrant acknowledgments. First, as a single-case report, the findings lack generalizability and require validation through larger controlled studies. Second, the absence of long-term follow-up precludes assessment of sustained functional outcomes or recurrence risks. Third, the multifaceted interventions (pharmacological, surgical, and rehabilitation) make it challenging to isolate the exclusive contribution of pulmonary rehabilitation to the patient’s recovery. Finally, subjective components in functional assessments (e.g., mMRC score and Borgs score) may introduce measurement bias, despite standardized tools being employed.

## 6. Conclusion

This case highlighted the critical role of integrated pulmonary rehabilitation in managing an elderly stroke patient with tracheostomy and TBPE. Early implementation of targeted respiratory muscle training, airway clearance techniques, and phrenic nerve pacing (combined with pharmacological therapy and pleural drainage) facilitated significant improvements in pulmonary function, accelerated pleural effusion resolution, and enabled successful decannulation. These outcomes emphasize the necessity of addressing respiratory dysfunction as a cornerstone of poststroke rehabilitation to mitigate complications and shorten ICU dependency. Clinicians should prioritize multidisciplinary rehabilitation protocols, particularly in high-risk populations, to optimize functional recovery. Future multicenter randomized trials are warranted to standardize interventions and evaluate their generalizability across diverse stroke subtypes.

## Acknowledgments

We are particularly grateful to Professor Li Hao from the Department of Pathophysiology, School of Basic Medical Science, Huazhong University of Science and Technology, for his guidance and assistance with our article.

## Author contributions

**Conceptualization:** Haitang Wei.

**Data curation:** Ying Huang.

**Formal analysis:** Jun Yang.

**Investigation:** Ying Huang.

**Supervision:** Lu Yang.

**Validation:** Haitang Wei.

**Visualization:** Tiecheng Guo.

**Writing – original draft:** Haitang Wei, Ying Huang.

**Writing – review & editing:** Lu Yang.

## Supplementary Material


