# Supplementary figures and images for: Pulmonary rehabilitation of a 72-year-old male with tracheostomy combined with unilateral tuberculous pleural effusion after cerebral infarction: A case report and literature review
Source: Medicine (Baltimore). 2025 Jul 18;104(29):e43360. doi: 10.1097/MD.0000000000043360 (PMC12282773; doi:10.1097/MD.0000000000043360)

## Slide 1
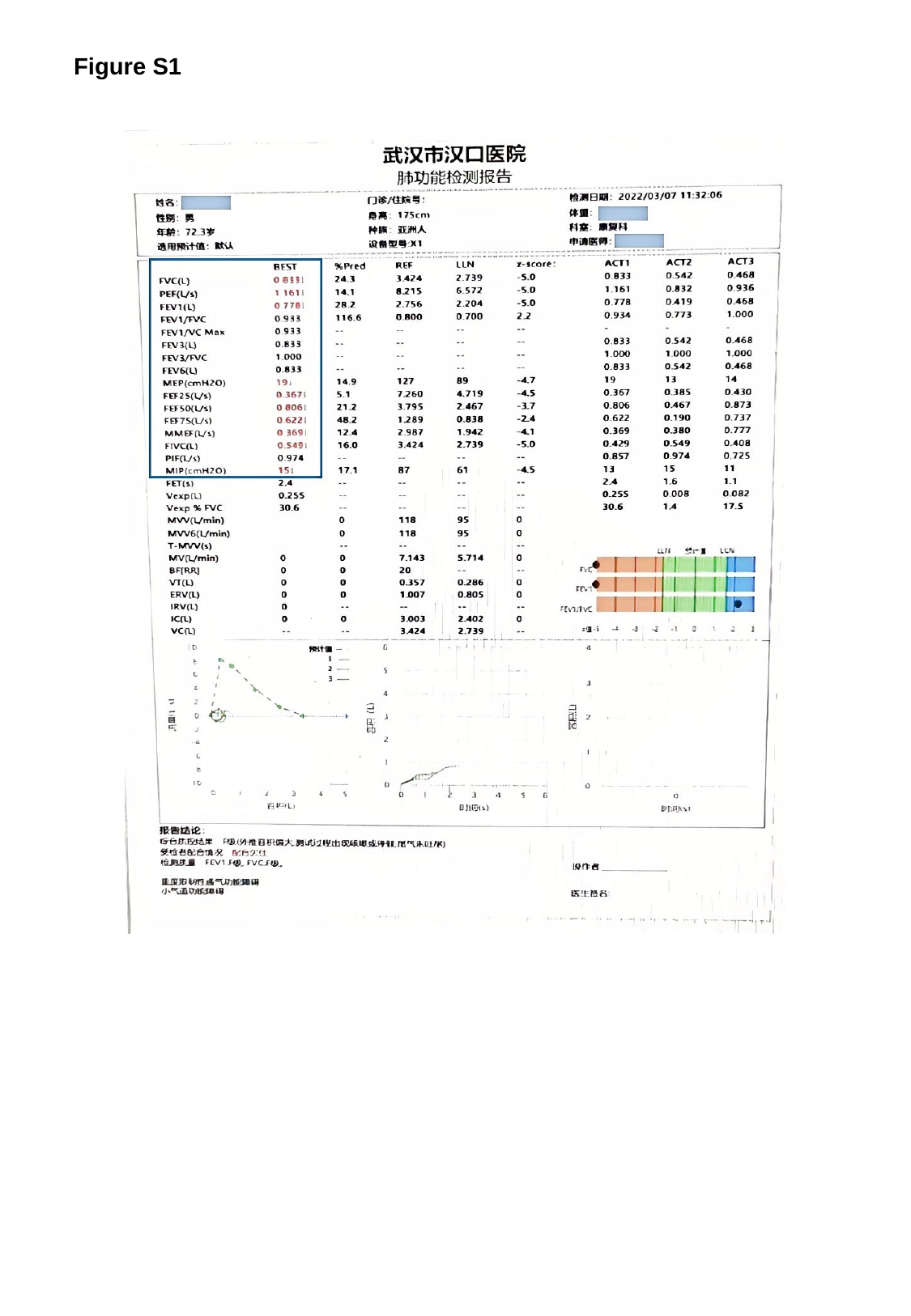

Figure S1

## Slide 2
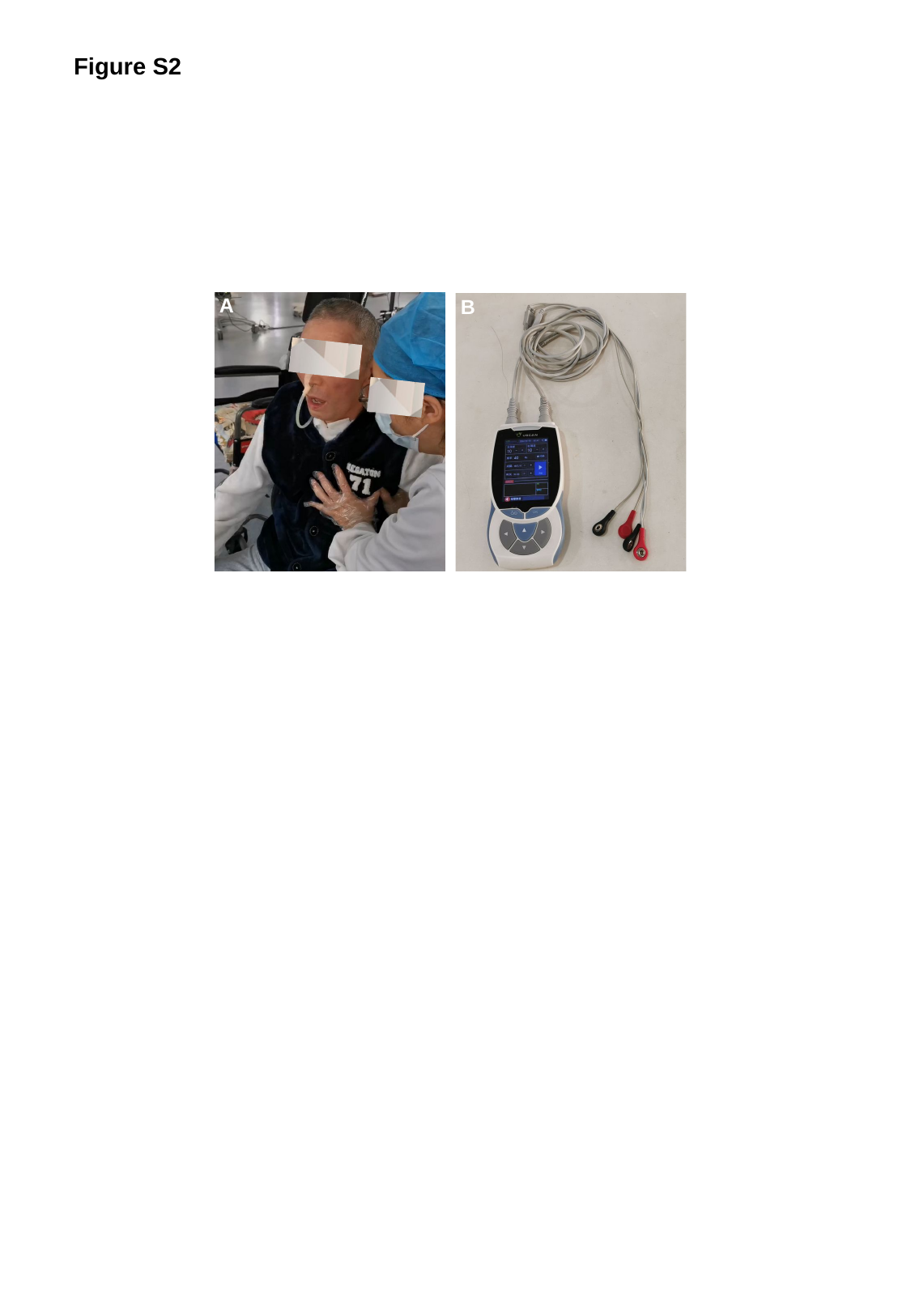

Figure S2
A
B

## Slide 3
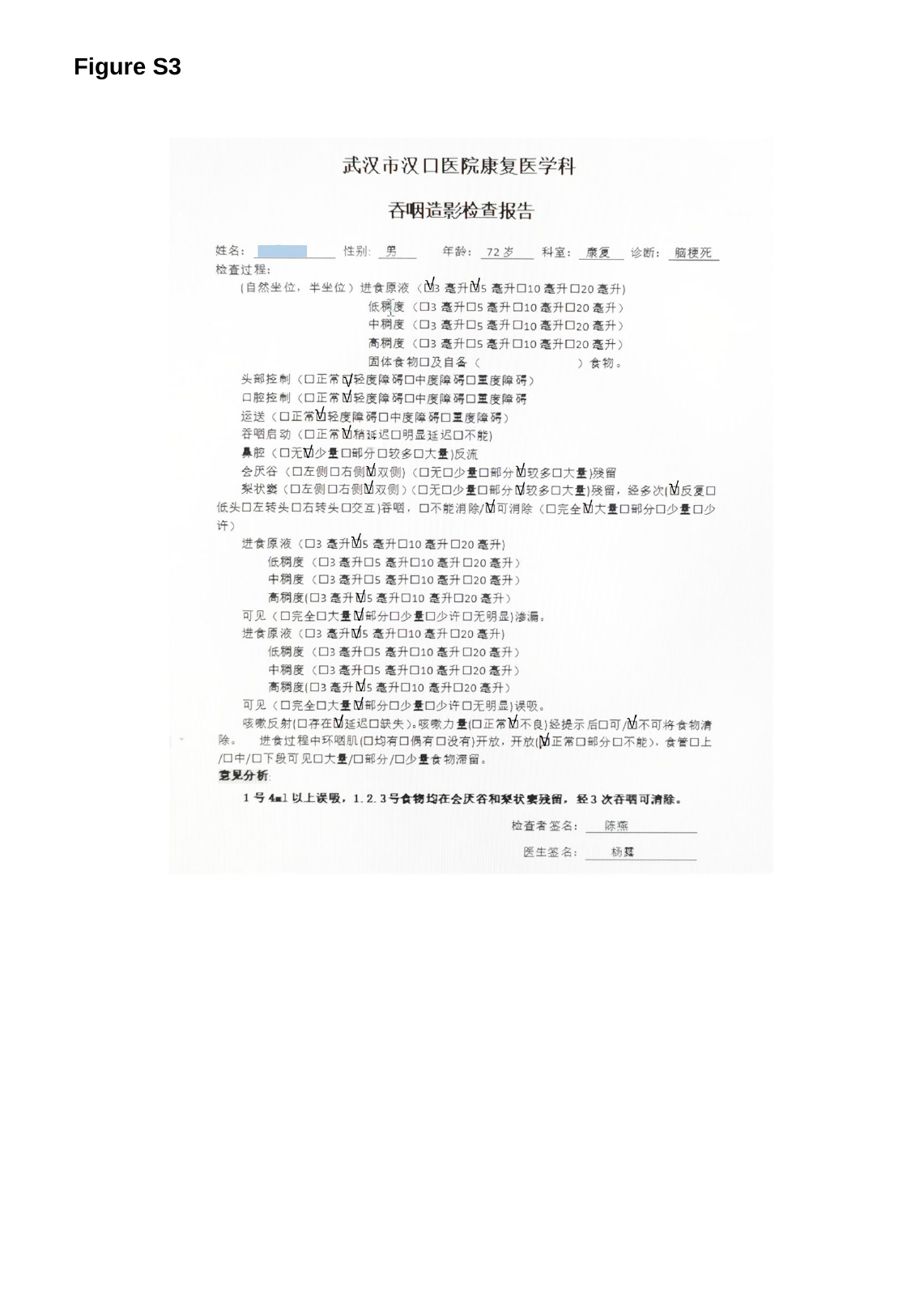

Figure S3

Supplement: Supplementary file 1 [file medi-104-e43360-s001.pptx]
