# Supplementary material for: Pulmonary rehabilitation of a 72-year-old male with tracheostomy combined with unilateral tuberculous pleural effusion after cerebral infarction: A case report and literature review
Source: Medicine (Baltimore). 2025 Jul 18;104(29):e43360. doi: 10.1097/MD.0000000000043360 (PMC12282773; doi:10.1097/MD.0000000000043360)
Supplement: Supplementary file 2 [file medi-104-e43360-s002.doc]

**Pulmonary Rehabilitation Training: Protocol Supplement**

**Respiratory Training**

During the training, the tracheostomy tube opening was temporarily sealed with medical tape, and vigilant monitoring of the patient's complexion, mental status, heart rate, oxygen saturation, and respiratory distress was conducted. Each session lasted for 20 minutes, twice daily.

① Abdominal breathing and pursed-lip breathing were practiced with an inspiratory to expiratory ratio ranging from 1:2 to 1:3 in order to reestablish respiratory pattern.

- **Abdominal breathing**

**Rationale**: Abdominal breathing utilizes a diaphragmatic-dominant breathing pattern to enhance diaphragmatic mobility, reduce accessory respiratory muscle recruitment, and improve alveolar ventilation efficiency by optimizing thoracic expansion.

**Procedure:**

**Positioning:** Assume an upright seated position with head-neck-trunk alignment to minimize postural interference with diaphragmatic movement.

**Inspiratory Phase:** Inhale slowly through the nose (5-8 seconds), focusing on abdominal protrusion while maintaining chest stability to ensure diaphragmatic descent and longitudinal thoracic expansion.

**Expiratory Phase:** Exhale fully via pursed-lip breathing (10-15 seconds), actively contracting abdominal muscles to elevate the diaphragm and reduce residual lung volume.

**Frequency:** 4-6 cycles/minute,3-5 minutes/session. Avoid hyperventilation by monitoring respiratory rate and SpO₂ (>88%) during training.

- **Pursed-Lip Breathing**

**Rationale:** Prolonged expiration (inspiratory-to-expiratory ratio 1:2-1:3) generates positive airway pressure, preventing small airway collapse and improving gas exchange by reducing air trapping and optimizing ventilation-perfusion matching.

**Procedure:**

**Expiratory Control:** Exhale through pursed lips (mimicking a whistling posture) for 2-3 times the inspiratory duration (e.g., 5-second inhalation → 10-15-second exhalation) to maintain bronchial patency.

**Resistance Monitoring:** Validate expiratory effort using a candle flame test, where the flame tilts without extinguishing at 15-20 cm from the lips, ensuring optimal expiratory resistance.

- Integration with abdominal breathing: Combine with abdominal breathing to establish a synergistic respiratory pattern—"abdominal expansion during inspiration + abdominal contraction with pursed-lip expiration"—enhancing tidal volume and oxygenation efficiency.

② Resistance training for inspiratory and expiratory muscles using a dual-valve breathing trainer(Fig. 4A), with incremental increases in respiratory resistance based on the patient's tolerance and adaptability during the training sessions.

- **Inspiratory/Expiratory Muscle Training Using a Dual-Valve Respiratory Device.**

**Rationale**: The device employs adjustable inspiratory/expiratory resistance valves to selectively strengthen inspiratory muscles (diaphragm, external intercostals) and expiratory muscles (abdominal muscles, internal intercostals), thereby enhancing muscle strength and endurance through targeted loading .

**Training Protocol**

**Initial Setup：**Select low resistance settings (inspiratory: 10-15 cmH₂O; expiratory: 5-10 cmH₂O).

Attach the mouthpiece and ensure an airtight seal to prevent air leakage.

**Inspiratory Muscle Training：**Exhale slowly to residual volume, then inhale deeply and slowly through the nose (≥3 seconds) against resistance until the pressure indicator reaches the target level.

Hold breath for 1-2 seconds, remove the mouthpiece, and perform pursed-lip exhalation (6-8 seconds). Repeat 10-15 cycles/set.

**Expiratory Muscle Training：**After deep inhalation, exhale forcefully through the mouthpiece (≥4 seconds) against expiratory resistance to activate abdominal and internal intercostal muscles.Perform 10-20 cycles/set with 30-second rest intervals between sets .

**Progression:** Assess tolerance weekly and gradually increase resistance (≤5 cmH₂O per session), targeting 30-50% of the patient’s maximal inspiratory pressure (MIP) .

- Integrate abdominal and pursed-lip breathing patterns to optimize neuromuscular coordination.

**Safety Monitoring**

**Safety thresholds:** Maintain SpO₂ ≥88% and limit heart rate increase to <20 bpm during training to mitigate respiratory muscle fatigue.

**Airway Clearance Therapy**

This treatment aided in the expectoration of sputum, reduced airway obstruction and mitigated pulmonary infection. Conducted for 30 minutes per session, twice daily.

**① Cough Training**(Fig. S2A).

The patient was guided to take a deep inspiration, hold breath for 1-2 seconds, and then coughed vigorously. The therapist applied manual abdominal compression during exhalation to enhance intra-abdominal pressure.

**② Manual Chest Vibration**.

The therapist's hands are positioned with fingers together and the palm cupped, delivering alternate percussive claps to the chest wall over the targeted drainage area.

**③Active Cycle of Breathing Techniques (ACBT).**

The patient performed his natural pattern of tidal breathing, followed by a voluntary breath hold at the end of inhalation for 3 seconds, and then forceful exhalation 1-2 times.

- **Breathing Control (Tidal Breathing)**

**Procedure:**Sit upright with relaxed shoulders; inhale slowly through the nose (2-3 sec), emphasizing diaphragmatic expansion.Exhale passively via pursed lips (4-6 sec) at a 1:2-1:3 inspiratory-to-expiratory ratio.

**Rationale:** Reduces accessory muscle recruitment, stabilizes respiratory rate (8-12 breaths/min), and minimizes airway collapse risk.

- **Thoracic Expansion with Breath Hold**

**Procedure:**Take a deep nasal inhalation to total lung capacity (5-8 sec), hold breath for 3 sec.Exhale passively; repeat 3-5 cycles.

**Rationale:** Breath-holding enhances collateral ventilation via alveolar interdependence, mobilizing secretions in obstructed airways.

- **Forced Expiratory Technique (FET)**

**Procedure:**

Low-volume huff: After normal inhalation (50% vital capacity), perform a sharp, open-glottis "huff" (4-6 sec) to clear peripheral secretions.

High-volume huff: Follow deep inhalation (80% vital capacity) with forceful huff and cough to expel central mucus.

Cycle 1-2 huffs followed by breathing control; repeat 3-5 cycles.

**Rationale:**Generates shearing forces to detach mucus with lower intrathoracic pressure (20-30 cmH₂O) than coughing, reducing dynamic airway collapse.

- **Safety & Monitoring**

Frequency: 2 sessions/day, 10-15 min/session.

Thresholds: Maintain SpO₂ ≥88%, heart rate increase <20 bpm.

**④Exhalation Positive Airway Pressure (EPAP) combined with High-Frequency Oscillation (HFO)training**(Fig.4B).

High-frequency oscillation aided in the mobilization of secretions, while EPAP supported lung expansion and prevented airway collapse.Utilizing a portable pulmonary function device, the airway pressure was incrementally adjusted from 3 cmH2O to 12 cmH2O based on the patient's tolerance.

- **Initial Parameters**

**EPAP:** Initiated at 3 cmH₂O and gradually titrated to 12 cmH₂O based on patient tolerance and SpO₂ levels (target ≥88%).Weekly increments of 2-4 cmH₂O, guided by real-time spirometric feedback (FEV1/FVC ratio) and hemodynamic stability (heart rate increase <20 bpm).

**HFO:** Applied at 5-15 Hz to enhance mucus mobilization.

- **Positioning**

Patients assumed an upright seated position with head-neck-trunk alignment to optimize diaphragmatic engagement.

- **Respiratory Pattern**

**Preparatory Phase:** Natural tidal breathing (8-12 cycles/min) for 2-3 minutes to stabilize respiratory rhythm.

**Inspiratory Phase:** Slow nasal inhalation to total lung capacity (5-8 seconds), followed by a 2-3-second breath-hold to enhance alveolar gas distribution.

**Expiratory Phase:** Exhale actively through a mouthpiece against EPAP resistance (3-12 cmH₂O) while the device concurrently delivered HFO to disrupt mucus adhesion.

- **Safety Monitoring**

Continuous SpO₂ monitoring (threshold ≥88%) and Borg dyspnea scale for subjective tolerance assessment.
